# Duct-to-Duct Anastomosis Versus Bilioenteric Anastomosis for Pediatrics Living Donor Liver Transplantation: A Systematic Review and Meta-Analysis

**DOI:** 10.7759/cureus.48108

**Published:** 2023-11-01

**Authors:** Beshoy Effat Elkomos, Philopateer Alkomos, Rao Junaid Saleem, Joseph Hanna, Guirgis Ebeidallah, Philobater B Awad, Basma Hassan, Ahmed Ghazal, Amr Abdelaal

**Affiliations:** 1 General and Emergency Surgery, Northwick Park Hospital, London Northwest NHS Trust, London, GBR; 2 Medicine, Ain Shams University, Cairo, EGY; 3 General Surgery, Wirral University Hospital, Cheshire, GBR; 4 Trauma, Orthopedics and Plastics Surgery, Salford Royal Hospital NHS Trust, Salford, GBR; 5 General Surgery, Ain Shams University, Cairo, EGY; 6 Orthopedics, Northwick Park Hospital, London Northwest NHS Trust, London, GBR

**Keywords:** biliary anastomosis, liver transplantation, pediatric ldlt, infant, biliary reconstruction

## Abstract

With an incidence exceeding 30%, biliary complications after pediatric liver transplantation remain a great challenge. In addition, the database includes numerous controversial papers about the safety of duct-to-duct anastomosis compared to Reux-en-Y hepaticojejunostomy for pediatric living donor liver transplantation (LDLT). We aim to compare the two techniques in pediatric LDLT by conducting a systematic review and meta-analysis. PUBMED, Web of Science, Scopus, and Cochrane Library were searched for eligible studies from 1989 to October 2022. According to our eligibility criteria, seven articles (561 pediatric LDLT) were included in our study. On one hand, DD anastomosis is associated with a higher rate of biliary stricture in comparison to RYHJ (OR: 2.47, 95% CI = 1.20-5.09, P = 0.01; I2 = 12%). On the other hand, the incidence of cholangitis was higher in RYHJ (OR: 0.10 95% CI = 0.01- 0.84, P = 0.03; I2 = 0%). However, there was no significant difference in the overall incidence of complications, leakage and mortality between the two groups (overall incidence of complication OR: 1.12, 95% CI = 0.34-3.68, P = 0.86; I2 = 62%), (Leakage OR: 2.22, 95% CI = 0.79-6.23, P = 0.13; I2 = 18%) and (Mortality OR: 2.53, 95% CI = 0.61-10.57, P = 0.30; I2 = 0%). In conclusion, with a lower incidence of cholangitis, an equal overall incidence of biliary complication, and the possibility of RY conversion in case of stricture, DD anastomosis offers a feasible, safe, and more physiological alternative to RYHJ for pediatric LDLT.

## Introduction and background

During the past few years, liver transplantation has become the standard of care for end-stage liver disease in children [1]. In addition to that, after the first successful pediatric living donor liver transplantation (LDLT) in 1989 and as a way to overcome deceased organ scarcity, the incidence of pediatric LDLT has increased dramatically [2,3]. Nevertheless, with an incidence exceeding 30%, biliary complications after pediatric liver transplantation remain a great challenge [4]. Furthermore, a third of those who develop these complications may die from biliary-related complications [5].

Numerous biliary reconstruction techniques have been adopted to decrease the incidence of these consequences, including Reux-en-Y hepaticojejunostomy, duct-to-duct, and duct-to-cystic duct [6]. However, RYHJ has been the standard for biliary reconstruction in pediatric patients. This is because biliary atresia is the most common cause of liver transplantation in pediatric patients [7]. On the other hand, duct-to-duct anastomosis is easier, and faster, with less manipulation of the small intestine, and facilitates the use of ERCP postoperative by maintaining normal bowel anatomy. In addition to that, according to some recent studies, duct-to-duct anastomosis in pediatric LDLT is feasible and provides similar outcomes to RYHJ [8,9].

Our aim was to give a clear, evidence-based recommendation for biliary reconstruction in pediatric LDLT by conducting a systematic review and a meta-analysis of the literature.

## Review

Patients and methods

Search Strategy

We searched the databases (PUBMED, Web of Science, Scopus, and Cochrane Library) from 1989 to October 2022 using a combination of these words: liver transplantation, living donor, pediatric, and biliary complications. Two authors (BE Elkomos and PE Alkomos) reviewed all articles independently. According to our inclusion and exclusion criteria, we started by reviewing the article based on the abstract followed by reading the full manuscript. In addition to that, an additional search was done using Google Scholar to detect any additional literature that could be included in our analysis.

Inclusion and Exclusion Criteria

Regarding the criteria of the included studies, they should be (1) retrospective, prospective, and randomized controlled trials. (2) Articles designed to detect the outcomes of pediatric LDLT. (3) Comparing the incidence of biliary complications between duct to duct, and RYHJ. (4) Articles written in English. And if the articles are case reports, case series, or reviews, designed to detect the outcomes of deceased donor liver transplantation in patients aged>18 years, and lacking a comparative group, they will be excluded.

Outcomes of Interest

Our outcome of interest was to detect the incidence of biliary complications (overall incidence of complications, biliary stricture, leakage, and cholangitis) and post-operative mortality for those who underwent duct-to-duct anastomosis and RYHJ.

Data Extraction

The following data were extracted for each of the included studies, data related to study design (author, year of publication, country of transplant, sample size, and follow-up period), patient details (age, sex, weight, ABO incompatibility, and GRWR), operative details (operative time, blood loss, cold ischemic time and warm ischemic time), biliary complications (biliary stricture, leakage, and cholangitis) and postoperative mortality. Two authors extracted the (BE Elkomos and PE Alkomos) independently.

Statistical Analysis

To conduct this meta-analysis, we followed the Cochrane Handbook for Systematic Reviews of Interventions [10], which is recommended by the Cochrane Collaboration. The pooled odds ratio (OR) and their 95% confidence interval were calculated for each of the biliary complications and postoperative mortality using a fixed effect model. However, if there was a significant heterogeneity (I2 > 40%), the random effect model was used. The results are considered statistically significant if the p < 0.05. All the calculations of this meta-analysis were done using Review Manager 5.4 (Cochrane Collaboration, Oxford, UK).

Results

Characteristics and Quality Assessment of Eligible Studies

**Figure 1:**
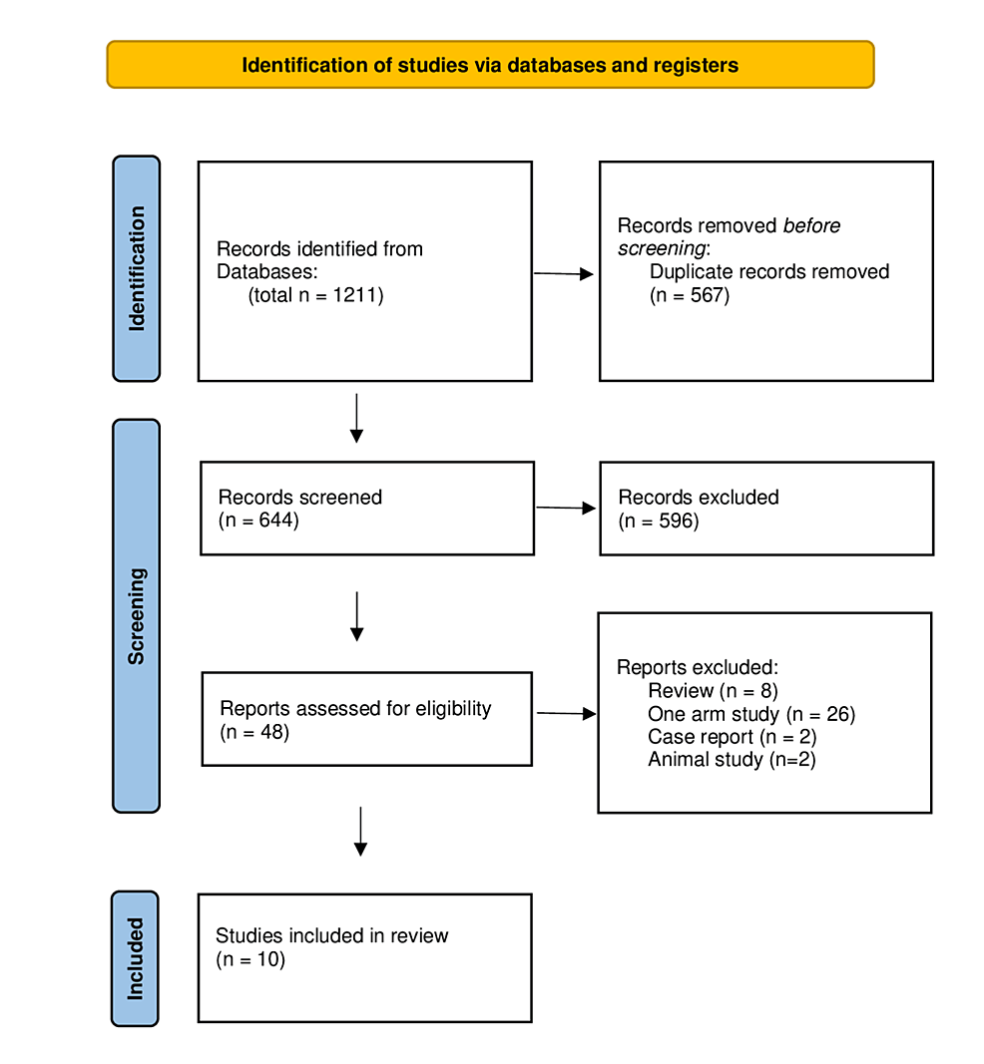
PRISMA flow diagram

As shown in the PRISMA flow diagram (Figure 1), 1,000 articles were selected by using this search string: liver transplantation, living donors, pediatric and biliary complications. After careful selection according to our eligibility criteria, seven articles [8,9,11-15] with a total of 561 pediatric patients who underwent LDLT. Six of the included studies were retrospective studies, one of them was prospective and no randomized controlled trial could be found. Four studies were conducted in Japan and the other three studies were done in China, Taiwan, and Turkey (Table 1).

**Table 1:** Basic data of the included studies (*) The results are presented as means and standard deviation. (**) The results are presented as median and range

Study (Year)	Country	Study period	Study design	Arm	Sample size	Sex (Male/Female)	Age (months)	Body weight (kg)	ABO incompatibility [n (%)]	Donor age (years)	GRWR	Cold ischemic time (min)	Warm ischemic time (min)	Operation time (min)	Blood loss (mL/kg)	Follow-up period (mo)
Shirouzu et al (2008) [11]	Japan	2001-2008	Retrospective	D-D	10	7/3	12.2 ± 9.9* (3–33) **	7.3 ± 2.4* (3.3–10) **	N/A	N/A	3.2 ± 0.9* (2.2–5.2) **	56.3 ± 40.5* (19–147) **	40.0 ± 6.7* (33–52) **	494.2 ± 71.8* (401–600) **	250.6 259.9 (35–915)	N/A
				RYHJ	20	N/A	N/A	N/A	N/A	N/A	N/A	N/A	N/A	N/A	N/A	N/A
Tanaka et al (2010) [12]	Japan	2005-2008	Retrospective	D-D	14	N/A	57.6 ± 46.7* (6-162) **	16.6 ± 7.3* (8-32) **	3 (21.4%)	37.8 ± 5.2* (32-47) **	2.1 ± 0.7* (1-3.4) **	41.1 ± 29.2* (16-125) **	37.9 ± 10.1* (23-58) **	557.7 ± 107.4* (428-846) **	133.7 ± 158.1 (8.3-513)	26.0 ± 11.5* (1.4-38) **
				RYHJ	46	N/A	46.5 ± 56.1* (1-215) **	14.9 ± 11.5 * (3.6-52) **	5 (10.9%)	36.2 ± 7.7* (20-62) **	2.5 ± 1.1* (.86-5.3) **	57.4 ± 35.2* (12-130) **	36.2 ± 11.0* (22-82) **	618.6 ± 200.0* (373-1290) **	138.6 ± 150.6 (2.2-705)	22.3 ± 10.7* (.33-44) **
Ando et al (2010) [13]	Japan	1998-2009	Retrospective	D-D	2	N/A	N/A	N/A	N/A	N/A	N/A	N/A	N/A	N/A	N/A	N/A
				RYHJ	47	N/A	N/A	N/A	N/A	N/A	N/A	N/A	N/A	N/A	N/A	N/A
Chok et al (2012) [14]	China	1993-2010	Prospective	D-D	4	N/A	N/A	N/A	N/A	N/A	N/A	N/A	N/A	N/A	N/A	N/A
				RYHJ	74	N/A	N/A	N/A	N/A	N/A	N/A	N/A	N/A	N/A	N/A	N/A
Chen et al (2013) [15]	Taiwan	2006-2011	Retrospective	D-D	17	N/A	N/A	N/A	N/A	N/A	N/A	N/A	N/A	N/A	N/A	N/A
				RYHJ	117	N/A	N/A	N/A	N/A	N/A	N/A	N/A	N/A	N/A	N/A	N/A
Yamamoto et al (2014) [8]	Japan	1999-2012	Retrospective	D-D	20	N/A	10.3 ± 9.8* (0.4 - 45) **	6.9 ± 2.0 (2.6-10.0) **	4 (20)	30.6 ± 3.4* (23-38) **	3.20 ± 0.70* (2.30-5.20) **	59 ± 46* (12-183) **	41 ± 7 (33-59) **	519 ± 114 * (355-770) **	N/A	N/A
				RYHJ	36	N/A	8.7 ± 4.2* (3-22) **	7.0 ± 1.4* (3.2-10.0) **	7 (19.4)	32.4 ± 7.6* (23-62) **	3.14 ± 0.59* (1.61-4.06) **	95 ± 48* (28-234) **	48 ± 26* (31-163) **	649 ± 135* (415-1004) **	N/A	N/A
Kilic et al (2020) [9]	Turkey	2009-2020	Retrospective	D-D	112	58/54	4.2 (0.29-17) ** Y	16.3 (4-55) **	N/A	N/A	1.94 (0.64-4) **	N/A	N/A	N/A	N/A	81.3 (12-128) **
				RYHJ	42	24/18	1.9 (0.08-14) ** Y	9.8 (3.5-44) **	N/A	N/A	2.62 (0.8-5) **	N/A	N/A	N/A	N/A	80.2 (12-128) **

Patient Outcomes

**Figure 2:**
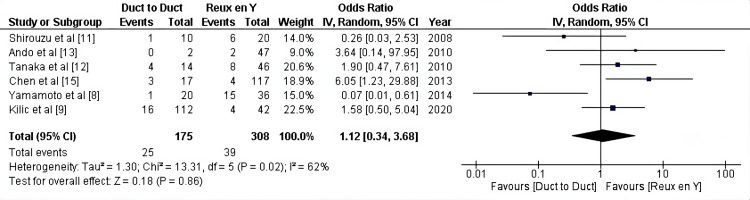
Overall incidence of complications

The overall incidence of biliary complications: According to 6 studies (483 patients), the overall incidence of biliary complications is similar in the two groups (OR:1.12, 95% CI = 0.34-3.68, P = 0.86; I2 =62%) (Figure 2).

**Figure 3:**
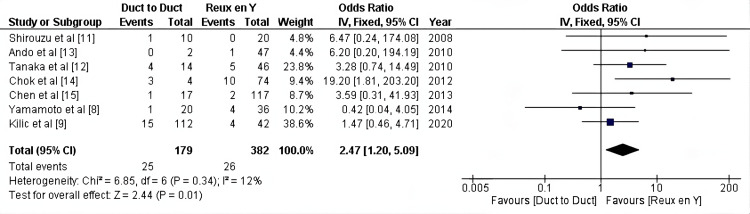
Biliary stricture

Biliary stricture: However, the pooled results of the seven included studies (561 patients) showed a higher rate of biliary stricture is associated with duct-to-duct anastomosis (OR:2.47, 95% CI = 1.20-5.09, P = 0.01; I2 =12%) (Figure 3).

**Figure 4:**
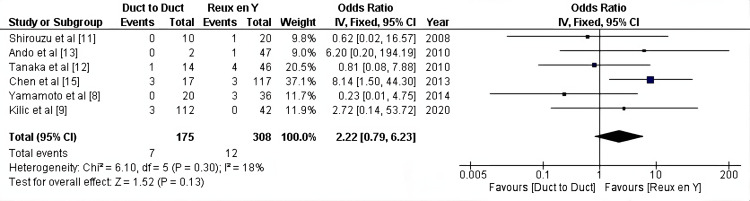
Leakage after liver transplant

Leakage: Regarding leakage after liver transplant as reported by six studies (483 patients), no significant difference in the incidence of leakage could be detected between the two groups (OR: 2.22, 95% CI = 0.79- 6.23, P = 0.13; I2 =18%) (Figure 4).

**Figure 5:**
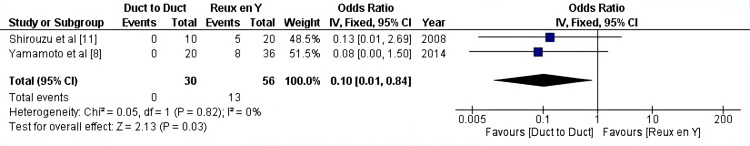
Increase in the rate of cholangitis

Cholangitis: Nevertheless, the pooled results of two studies (86 patients) showed a significant increase in the rate of cholangitis is associated with RYHJ (OR: 0.10 95% CI = 0.01- 0.84, P = 0.03; I2 =0%) (Figure 5).

**Figure 6:**
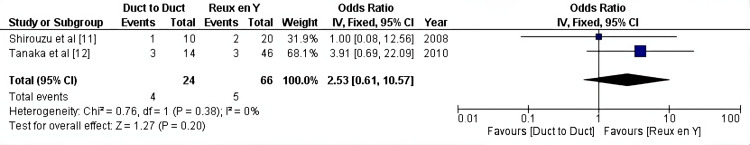
Post-operative mortality

Mortality: Turing to post-operative mortality, no remarkable difference could be detected between duct-to-duct and RYHJ as reported by two studies (90 patients) (OR:2.53, 95% CI = 0.61-10.57, P = 0.30; I2 =0%) (Figure 6).

Discussion

According to our study, on one hand, duct-to-duct anastomosis is associated with a higher incidence of biliary structure in comparison to RYHJ. On the other hand, RYHJ has a higher rate of cholangitis. However, the two techniques of biliary reconstruction have a similar overall incidence of biliary complications, leakage and postoperative mortality.

DD anastomosis has become widely used in adult-living donor liver transplants even in the presence of multiple bile duct anastomosis [16]. The advantage of DD over RYHJ includes less manipulation of the small bowel, no intestinal anastomosis and preservation of the valvular mechanism of the sphincter of Oddi decreasing the incidence of cholangitis.

However, in pediatric LDLT RYHJ is still the gold standard anastomosis because of the small size of the duct especially in children with low body weight and most of the cases of pediatric liver transplantation are biliary atresia. In addition to that, there are a lot of controversial topics related to the safety of DD anastomosis in pediatric LDLT. According to Sakamoto et al., 47% of patients who had DD anastomosis experienced complications and 26% of patients were converted to RY [17]. In addition to that, in 2010, Tanaka et al. reported a higher incidence of complications related to DD anastomosis in comparison to RYHJ (28.6% vs 17.4%) [12]. On the other hand, as reported by another Japanese study, DD anastomosis is a feasible option for biliary reconstruction even in low child weight with no statistically significant difference between the two types of reconstruction in terms of the incidence of leakage and stricture [8]. The pooled results of the included studies showed no statistically significant difference in the overall incidence of biliary complication between the two types of anastomosis.

To begin with biliary stricture after pediatric liver transplantation, this complication can occur within 2 years of transplantation [8]. Shirouzu et al. [11] and Yamamoto et al. [8] Reported that no statistically significant difference in the incidence of biliary stricture between the two groups. However, in line with what Tanaka et al. [12] and Chok et al. [14] reported, our study showed that the incidence of biliary stricture was higher in the case of D-D anastomosis. The exact reason for this is not clear. However, according to Tanaka et al. [12], this might be because of the growth of the graft which is associated with an increase in the size of the graft that will lead to stretch of the anastomosis leading to fibrosis at the anastomosis site. Moreover, according to Kilic et al. [9], the management of DD stricture using ERCP could be hazardous because of the small size of the duct. However, in the case of DD anastomosis, operative conversion to RY is still an option after PTC decompression.

Regarding cholangitis after transplantation, Shirouzu et al. [11] and Yamamoto et al. [8] reported 0% incidence of complication in the case of DD anastomosis, however, the incidence of cholangitis in RYHJ was up to 22% of the cases. In line with that, the pooled results of the included studies showed a higher incidence of cholangitis in case of RYHJ. This has been explained by the reflux of the intestinal content to the biliary tree in case of RYHJ due to the bypass of the sphincteric mechanism of the sphincter of odd [11].

According to 60 pediatric LDLT operated in Japan, no statistically significant difference between the two groups in terms of the incidence of leakage (DD vs RYHJ 0% vs 6.5% P=NS) [12]. On the other hand, according to 154 pediatric LDLT in Turkey, the incidence of leakage is higher in case of DD anastomosis (2.7 % vs 0% for DD vs RYHJ); however, the value was not statistically significant [9]. In this study, no significant difference in the incidence of leakage between the two types of biliary reconstruction.

To our knowledge, it is the first meta-analysis to compare DD and RYHJ in pediatric LDLT. In addition to that, all the studies in the database comparing the two modalities were included. However, we have to admit the presence of some limitations in our study; the effect of using a stent in biliary reconstruction was not included. However, we addressed the use of biliary stents in liver translation in another research paper [18]. Moreover, no RCT was found to be included in our study.

## Conclusions

In comparison to RYHJ, DD anastomosis is more physiological leading to a lower incidence of cholangitis. Despite having a similar overall incidence of complications, DD anastomosis offers a second line of operation by conversion to RYHJ. Thus it can be said that DD anastomosis offers a feasible and safe alternative to RYHJ for pediatric LDLT.
